# Intraoperative fine needle aspirations - diagnosis and typing of lung cancer in small biopsies: challenges and limitations

**DOI:** 10.1186/s13000-016-0510-6

**Published:** 2016-07-07

**Authors:** Christian Biancosino, Marcus Krüger, Ekkehard Vollmer, Lutz Welker

**Affiliations:** Department of Cardiac, Thoracic, Transplantation and Vascular Surgery, Medical School Hannover, Hannover, Germany; Department of Thoracic Surgery, Helios University Hospital Wuppertal, University Witten/Herdecke, Heusnerstraße 40, 42115 Wuppertal, Germany; Department for Clinical and Experimental Pathology, Research Center Borstel Airway Research Center North, Member of the German Center for Lung Research, Parkallee 35, Borstel, 23845 Germany; Cytology Laboratory, LungenClinic Grosshansdorf Airway Research Center North, Member of the German Center for Lung Research, Woehrendamm 80, Großhansdorf, 22927 Germany

**Keywords:** Fine needle aspiration cytology, Lung cancer typing

## Abstract

**Background:**

Due to therapeutic implications with regard to both efficiency and safety of chemotherapy agents it is important to differentiate between subtypes of NSCLC. Up to today we experience a continuous reservation regarding the use of fine needle aspiration cytology. The aim of the present study is to estimate the value of cytologic criteria for lung cancer typing on small biopsies independent from all possible technique failures.

**Methods:**

Between January 1997 and December 2008 760 intraoperative FNAC- (fine needle aspiration cytology) specimens from 702 patients have been examined. Cytologic evaluation and immediate communication of results to the surgeons followed. Afterwards, intraoperative cytologic findings were compared with final histologic diagnoses of the resected specimens.

**Results:**

Intraoperative cytologic analysis yielded a sensitivity of 94.8 %, a specificity of 98.8 %. An overall positive predictive value of 99.8 % with respect to final histologic analysis of primary lung cancer was achieved. The highest value could be reached for adenocarcinomas, followed by carcinoids and squamous cell carcinomas.

**Conclusions:**

Lung cancer typing according to cytologic criteria is feasible and accurate as well as comparable with results of histologic analysis on small specimens. Herewith, clinicians can come up to the increasing demands on minimally invasive harvested specimens with regard to therapeutic implications.

## Background

Most lung cancer patients present in advanced inoperable stages and the diagnosis is based on small specimens, like small forceps or needle biopsies and/or cytology. In addition to new biopsy techniques, especially in combination with ultrasound, a paradigm shift has occurred for both pathologists and clinicians [1, 2].

With respect to this shift there is an increased significance of cytologic aspects regarding biopsy procedures themselves as well as morphologic analysis [3].

Furthermore, due to important therapeutical implications e.g. concerning chemotherapy regimens in contrast to former times it is not only important to distinguish between SCLC and NSCLC but also between their subtypes of squamous cell carcinomas and adenocarcinomas. Several chemotherapy agents have become part of clinical daily routine with indication contingent on definition of subtype, with regard to both efficiency and safety [4]. For example, EGFR mutations, which are predominantly found in adenocarcinomas, are a prerequisite for the application of EGFR tyrosine kinase inhibitors. Other current agents like bevacizumab and pemetrexed are only approved for patients with non-squamous cell carcinomas due to specific toxicity and effectiveness.

In the 2004 WHO classification of lung tumors, cytology was addressed for the first time, but subclassification on small biopsies was not taken into account.

Methodical difficulties of tumor typing on FNAC specimens are comparable with those on small biopsies like core and transbronchial biopsies.

FNAC, especially in combination with ultrasound is usually the first choice of diagnostic procedures in lung cancer. Its utility in the diagnosis of endobronchial, peripheral, and mediastinal lesions has been reported and confirmed in various clinical reports [5, 6]. Most current papers put their emphasis on sensitivity, specificity and diagnostic reliability of FNAC in the diagnosis of malignant tumors. The specificity in these reports is usually very high.

The most important factors that influence FNAC include localization of lesions, length and diameter of the needle as well as the individual kind of biopsy procedure (percutaneous FNA, CT- or ultrasound guided FNA, TBNA (transbronchial needle aspiration), EBUS- or EUS-FNA) [7].

However, in practice there are continuous reservations using the results of fine needle aspiration cytology. In this direction the general problems will be well illustrated by the question by Langer et al. “Can we use cytologic diagnosis?” and its reply in JCO by Fischer et al. [8, 9].

In practice faults in biopsy techniques are generally interpreted as failure of cytology. Such a wholesale judgement analysis prevents a correct assessment of the methodical limitation of different biopsy techniques as well as the critical judgement of cytological procedures.

FNAC during thoracic surgery is a suitable model to determine the efficiency of lung cancer typing on small biopsies. The possibility of minimizing sampling errors is quite different regarding the individual biopsy procedure and accounts for the different values of sensitivity [10].

Interestingly, intraoperatively harvested FNAC specimens are less influenced by technique of biopsy than the above mentioned. For example, due to direct digital identification of tumors, sampling errors can be nearly excluded. Furthermore, there is comprehensive feedback of the biopsy by following on site cytologic analysis and by final histologic diagnosis of the resected specimen.

Thus, methodic limitations such as incorrect needle placement, large amounts of fibrous material, scanty cellularity of tissue, as well as improper smear preparation, which are commonly regarded to account for most false negative FNAC findings on ultrasound biopsies, can be avoided intraoperatively.

In contrast to false negative FNAC findings, a false-positive diagnosis is generally related to the interpretation of the aspirates. In such cases a cytologist attempts to interpret scant material that contains only few abnormal cells. However, intraoperatively harvested FNAC specimens contain large numbers of cancer cells and small tissue fragments.

The aim of the present study is to estimate the value of cytologic criteria for lung cancer typing on small biopsies independent from all possible technique failures.

## Methods

At our hospital, a total of 760 FNAC-specimens from 702 consecutive patients (454 males, 248 females; mean ± SD age 62.0 ± 9.8 years) were evaluated between January 1997 and December 2008.

Included were all patients with an extensive, preoperative, bronchoscopic work up with EBUS and/or EUS fine needle aspirations in whom the dignity of tumor could not be specified.

For intraoperative biopsy procedures Yale-Spinal-Needles by Becton Dickinson GmbH (Heidelberg, Germany) were used. All surgeons and nurses in the OR were trained in optimal smear preparation.

### Slide preparation

An optimal smear preparation allows the distribution of well-preserved cells and small tissue fragments on the slide.

It was important to prepare a thin and uniform smear with well-preserved cells and small tissue fragments. Spraying, squeezing artifacts and blood clots were avoided, as all this distorts the architecture of cell clusters and may conceal microscopic details. For each bioptic procedure the needle was inserted at least two times. Air-dried smears were fixed in 95 % methanol and stained with Giemsa (staining solution according to Schlüter).

Following main cytologic features for smear preparations for the different types of lung cancer were used:

- Squamous Cell Carcinoma: Single pleomorphic cells and syncytial sheets of cells with sharp cytoplasmic outlines and abnormal cytoplasmic thinning manifested as caudate and spindle cells. Nuclei are irregularly shaped and centrally located. Chromatin is coarsely granular. Keratinized squamous ghosts in a background of debris and blood.
- Adenocarcinoma: Large, three-dimensional cell groups appear in spherical or oval clusters, and acini with single cells. Individual cubical or columnar configured tumor cells, with basophilic or vacuolated cytoplasm. One or more prominent nuclei are present. Nuclei are eccentrically placed. Chromatin is finely granular to dusty.
- Large-Cell Carcinoma: A mixture of large, single cells and syncytial groups with large, and round to oval-shaped nuclei can be seen. Chromatin is intermediate between squamous carcinoma and adenocarcinoma.
- Small-Cell Carcinoma: Cells occur in small clusters, clumps, and single cells with intercellular moulding. Apoptotic cells, mitotic figures, nuclear moulding and single-file pattern are frequently encountered. Nuclei vary from round to very irregular with salt-and-pepper chromatin (PAP). The background is filled with individual cell necrosis seen as small, dark, pyknotic nuclei [11].
- Carcinoid tumor: Cells appear in small monolayer clusters, and single cells without intercellular moulding. No apoptotic cells and mitotic figures can be observed. Nuclei vary from round to uniformly oval. Sometimes small protuberances can be observed. As in other neuroendocrine tumors a salt-and-pepper chromatin is typical.

### Data analysis

Sensitivity, specificity and diagnostic reliability were computed as usual.

## Results

In total 702 consecutive cases were analyzed. Adenocarcinoma was the most frequent diagnosis and made up 47.9 of all cases. The other major diagnoses in descending order of frequency were squamous cell carcinomas (20.1 %), large cell/large cell neuroendocrine carcinomas (12.8 %), and benign lung processes (12.8 %) (Table 1).

**Table 1:** Results of intraoperative fine needle aspiration cytology of malignant lung tumors and benign lung lesions. Number of all histological diagnoses compared with corresponding and different cytological findings

Cytological findings	Final histological diagnosis								
	SQC	SCLC	ADC	ADC/SQC	LC/LNC	Carcinoid	Other	Benign	Total
SQC	98	0	19	4	11	0	0	0	132
SCLC	1	7	1	0	3	1	0	0	13
ADC	7	0	210	3	18	1	0	0	239
LC/LNC	13	1	48	0	39	1	1	0	103
Carcinoid	0	0	3	0	1	13	0	0	17
Other	0	0	1	0	2	0	0	1	4
Susp.	2	0	7	0	2	0	0	1	12
Benign	6	0	14	1	5	2	0	84	112
Total	127	8	303	8	81	18	1	86	632

*Abbreviations*: *SQC* Squamous cell carcinoma, *SCLC* Small Cell Lung Carcinoma, *ADC* Adenocarcinoma, *ADC/SQC* Adeno-squamous carcinoma, *LC/LNC* Large Cell Carcinoma/Large Cell Neuroendocrine Carcinoma, *Other* Other malignant tumor, *Susp*. tumor suspicious findings

Cytologically 126 cases were classified as benign, 563 as malignant and 13 cases suspicious of malignancy.

**Fig. 1:**
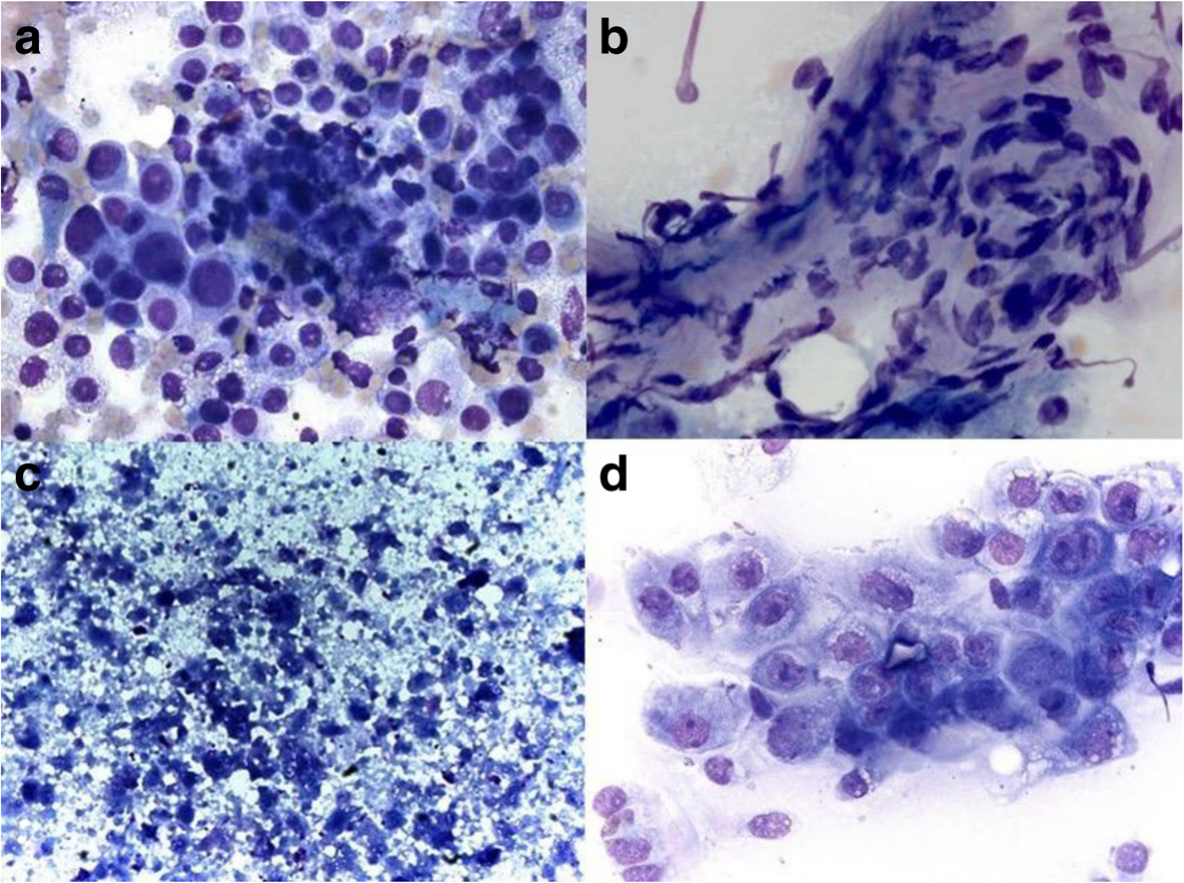
Intraoperative Fine Needle Aspirations in cases of false negative cytological diagnosis. Tumor cells of a finally high differentiated adenocarcinoma (bronchiolo-alveolar cell type, **a** Giemsa, 630x). Infiltration of malignant mesenchymal cells of a fibrosarcoma. **b** Giemsa staining of tumor in high magnification (630x). Necrotic area from the center and squamous epithelial cell dysplasia in the surrounded neighborhood of a finally squamous cell carcinoma (**c** and **d**)

Among the cytologically benign cases 37 were determined malignant in final histologic analysis. 28 cases of this collective were malignant lung tumors in the final interpretation, 9 cases were assessed as non primary lung tumors (Tables 2 and 3). Cytologically, in 11 cases unsuspicious lung parenchyma, lymph nodes or bronchial epithelium was seen, in 8 cases a severe inflammation, in 2 cases only blood fragments could be identified. In 5 cases of highly differentiated adenocarcinomas it was not possible to differentiate between reactively altered pneumocytes type II, adenomatous hyperplasia or highly differentiated adenocarcinomas. In 3 cases it was not possible to specify the dignity of tumors later determined as malignant mesenchymal tumors (Fig. 1)

**Table 2:** Results of intraoperative fine needle aspiration cytology of malignant lung tumors and benign lung lesions. Description of the 28 false negative cases

Cytological findings	Final histological diagnosis				
	SQC	ADC	LC/LNC	Carcinoid	Total
Normal tissue	2	6	0	2	10
Necrosis	0	1	2	0	3
Restenotic pneumonia	4	1	1	0	8
High differentiated tumors	0	5	0	0	5
Dysplasie	1	0	0	0	1
Blood	0	1	5	0	1
Total	7	14	8	2	28

*Abbreviations*: *SQC* Squamous cell carcinoma, *ADC* Adenocarcinoma, *LC/LNC* Large Cell Carcinoma/Large Cell Neuroendocrine Carcinoma

**Table 3:** Results of intraoperative fine needle aspiration cytology of malignant tumors (other than primary lung cancer). Description of the 9 false negative cases

Cytological findings	Final histological diagnosis		
	SAR	ADC	Total
Normal tissue	1	2	3
Benign mesenchymal lesion	3	0	3
Blood	1	2	3
Total	5	4	9

*Abbreviations*: *SAR* Sarcoma, *ADC* Adenocarcinoma

One patient with a chronic inflammatory process and one case of lipomatous atrophic thymic tissue were judged cytologically as malignant and suspicious of malignancy, respectively. In summary, rapid intraoperative cytolgic evaluation yielded a sensitivity of 94.8 %, a specificity of 98.8 %.

The communication to the surgeon included the cytological judgement (benign, malignant, suspicious) and in case of malignancy the classification as either primary or metastatic lung tumor and concerning the latter its origins. Additionally, a detailed evaluation was given in case of benign lesions or lesions suspicious of malignancy in order to facilitate surgical decision making.

Using conventional Giemsa staining only, an overall positive predictive value of 99.8 % with respect to final histologic analysis of primary lung cancer was achieved. The highest value could be reached for adenocarcinomas (88 %), followed by carcinoids (77 %) and squamous cell carcinomas (74 %). Diagnosis of adeno-carcinomas had lower sensitivity than squamous cell carcinomas (71 % versus 78 %, respectively). The specificity was lower as well (91 % versus 93 %, respectively) (Table 4).

**Table 4:** Calculated sensitivity, specificity, false positive, false negative and true positive rates, negative and positive predictive values (tumor suspicious lesions excluded)

	All	SQC	SCLC	AD	LC	Carcinoid
Sensitivity	94.8 %	78.4 %	87.5 %	70.9 %	49.4 %	72.2 %
Specificity	98.8 %	93.1 %	99.0 %	91.0 %	88.2 %	99.3 %
False positive rate	1.2 %	6.9 %	1.0 %	9.0 %	11.8 %	0.7 %
False negative rate	5.2 %	21.6 %	12.5 %	29.1 %	50.6 %	27.8 %
Negative predective value	75.0 %	94.5 %	99.8 %	77.4 %	92.3 %	99.2 %
Positive predective value	99.8 %	74.2 %	53.8 %	87.9 %	37.9 %	76.5 %

When all cytological criteria of lung cancer types were fulfilled levels for sensitivity and specificity were much higher especially for squamous cell carcinomas and adenocarcinomas (Tables 5 and 6).

**Table 5:** Results of intraoperative fine needle aspiration cytology of malignant lung tumors (cytological criteria for lung cancer typing fulfilled) and benign lung lesions. Number of all histological diagnoses compared with corresponding and different cytological findings

Cytological findings	Final histological diagnosis								
	SQC	SCLC	ADC	ADC/SQC	LC/LNC	Carcinoid	Other	Benign	Total
SQC	47	0	9	1	6	0	0	0	63
SCLC	0	3	1	0	3	0	0	0	7
ADC	1	0	107	1	3	0	0	0	112
LC/LNC	0	0	1	0	1	0	0	0	2
Carcinoid	0	0	3	0	1	13	0	0	17
Other	0	0	0	0	1	0	0	0	1
Susp.	0	0	1	0	0	0	0	0	1
Benign	6	0	14	1	5	2	0	84	112
Total	54	3	136	3	20	15	0	84	315

*Abbreviations*: *SQC* Squamous cell carcinoma, *SCLC* Small Cell Lung Carcinoma, *ADC* Adenocarcinoma, *ADC/SQC* Adeno-squamous carcinoma, *LC/LNC* Large Cell Carcinoma/Large Cell Neuroendocrine Carcinoma, *Other* Other malignant tumor, *Susp*. tumor suspicious findings

**Table 6:** Calculated sensitivity, specificity, false positive, false negative and true positive rates, negative and positive predictive values (tumor suspicious lesion excluded)

	All	SQC	SCLC	ADCa	LC	Carcinoid
Sensitivity	87.8 %	87.0 %	100.0 %	79.3 %	33.3 %	86.7 %
Specificity	100.0 %	93.8 %	98.7 %	97.2 %	99.7 %	98.7 %
False positive rate	0.0 %	6.2 %	1.3 %	2.8 %	0.3 %	1.3 %
False negative rate	12.2 %	13.0 %	0.0 %	20.7 %	66.7 %	13.3 %
Negative predective value	75.0 %	97.2 %	100.0 %	86.1 %	99.4 %	99.3 %
Positive predective value	100.0 %	74.6 %	42.9 %	95.5 %	50.0 %	76.5 %

In addition to primary lung tumors secondary pulmonary lesions were documented to create a comparable overall accuracy. Best results could be shown for malignant melanomas, adenocarcinomas and large cell carcinomas (Table 7).

**Table 7:** Results of intraoperative fine needle aspiration cytology of malignant tumors (other than primary lung cancer). Number of all histological diagnoses compared with corresponding and different cytological findings

Cytological findings	Final histological diagnosis									
	SAR	SQC	ADCa	LCA	Lym	Thym	Mel	Other	Benign	Total
SAR	1	0	0	0	0	0	0	0	0	1
SQC	0	3	1	0	0	0	0	0	0	4
ADCa	0	0	27	1	1	0	0	0	0	29
LCa	0	2	4	4	0	1	0	0	0	11
Thym	0	0	0	1	1	0	0	0	0	2
Mel	0	0	0	0	0	0	4	0	0	4
Other	0	0	0	0	2	1	0	1	0	4
Lym	0	0	0	0	0	0	0	0	0	0
Benign tumor	5	0	4	0	0	0	0	0	5	14
Susp.	1	0	0	0	0	0	0	0	0	1
Total	7	5	36	6	4	2	4	1	5	70

Abbreviations: SAR-Sarcoma, SQC - Squamous cell carcinoma, ADC - Adenocarcinoma, LCA - Large Cell Carcinoma, Lym – Hodgkin-/Non Hodgkin-Lymphoma, Thym-Thymoma, Mel- Melanoma, Other - Other malignant tumor

Regardless of high specificity a correct subclassification of benign lesions by rapid cytologic assessment was not as accurate as the typification of malignant tumors.

## Discussion

In recent years our concepts of lung cancer have undergone a revolution.

Due to therapeutic implications with regard to both efficiency and safety of several chemotherapy agents it is not only important to distinguish between SCLC and NSCLC but also to give information about the specific subtype of NSCLC [4]. In contrast to Edward’s et al. postulation this subtyping is not only possible but indispensable [12]. Although from our point of view the WHO criteria cannot be consulted for the analysis of cytologic specimens, a percentage of 40 % or more regarding non-small cell lung cancer - not other classified (NSCLC-NOS) seems no longer acceptable.

As most lung cancer patients are seen in advanced inoperable stages nowadays the diagnosis is primarily based on small biopsy or cytology specimens. The advantage of cytologic diagnosis is that it is based on small specimens that can be harvested easily by minimally invasive procedures like EBUS-FNA, TBNA or EUS.

Unfortunately, a histologic confirmation of these cytologic diagnoses is missing. Several studies have confirmed the excellent accuracy of cytology regarding differentiation between SCLC and NSCLC [13, 14].

The majority of the published data on techniques like EBUS, EUS or percutaneous FNA analyze the sensitivity and specificity of FNA with regard to lung cancer in general. When it comes to the subtyping of non-small cell lung cancer by means of cytological procedures, however, comprehensive reviews on large collectives are rare [7, 15].

FNAC during thoracic surgery is a suitable model to determine the efficiency of lung cancer typing on small biopsies. This study represents an assessment of independent morphologic cytology criteria based on a large collective.

Altogether, our intraoperative cytologic assessment of benign and malignant tumors yielded a sensitivity of 94.8 %, a specificity of 98.8 %.

The correct subclassification of benign lesions by rapid cytologic assessment was not as accurate as the typing of malignant tumors.

Generally speaking intraoperatively harvested fine needle aspiration biopsies are an excellent model to investigate the difficulties involved in the morphologic analysis of rapid evaluation and the evaluation of cytologic criteria for the identification of tumor typing without supplementary immunocytochemical procedures.

The fundamental histologic evaluation criterion for these two-dimensional samples is the detection of invasive and destructive tumor growth. In case of small biopsies, punch biopsies and tumor fragments, however, this method is much less effective. If invasive and destructive tumor growth cannot be detected evaluation is based only on a limited number of cytologic criteria.

In contrast to histology, the cytologic diagnosis is based on the evaluation of single cells, cell clusters and small, frequently three dimensional tissue fragments. Preserved tissue architecture is not essential but mostly also available for cytologic assessment. However, cytologic diagnosis is primarily based on thorough evaluation of a variety of different cytologic criteria on these single cells, cell clusters and small, frequently three dimensional tissue fragments.

In general, the accuracy of tumor diagnosis from frozen sections is very high. Xu et al. reported a sensitivity and specificity of 100 % in the diagnosis of pulmonary nodules [15]. However, with regard to rare diseases, it remains doubtful if only 229 cases, which is comparable to most studies in this field, are sufficient to draw substantiated or statistically significant conclusions [16, 17]. When considering a broad spectrum of diseases and a sufficient case load as achieved in the study by Hwang et al., more than 24 (0.54 %) false positive, 65 (1.47 %) false negative and 30 (0.77 %) deferred diagnoses are documented [18]. Our results are comparable to those shown by Orki et al. [19].

Overall, cytologic procedures are nearly as accurate as frozen section but they have limitations especially in tissues with sparsely scattered cells and with abundant stromal components. Nevertheless, an accurate evaluation regarding the distribution of single cells and the architecture of three dimensional tissue fragments allowed for valuable conclusions on the assumed corresponding tissue texture *in situ* due to their correlating arrangements. In squamous cell carcinoma, for example, a multilayered tumor growth and keratinization are predominantly observed, whereas in other tumor types, glandular or papillary as well as neuroendocrine features were predominantly found. In the majority of cases a differentiation between squamous cell and adenocarcinoma can already be achieved by morphology. The difficulty arises in samples which are poorly differentiated, scant or poorly preserved.

Although during rapid intraoperative evaluation further cuts cannot be produced and immunohistochemical analyses can only be performed to a certain extent, a high accuracy level regarding the typing of primary and secondary malignancies seems to be possible [20].

For primary lung cancers we were able to achieve an overall positive predictive value of 99.8 % with respect to final histologic analysis. The highest value could be reached for adenocarcinomas (88 %), followed by carcinoids (77 %) and squamous cell carcinomas (74 %). Diagnosis of adenocarcinomas had lower sensitivity than squamous cell carcinomas (71 % versus 78 %, respectively). The specificity was lower as well (91 % versus 93 %, respectively).

The rather low levels especially for adenocarcinomas may be due to several reasons. First of all it is well known that poorly differentiated tumors are difficult to classify cytologically.

Levels for sensitivity and specificity were much higher, squamous cell carcinomas and adenocarcinomas (Table 6), when tumors in which not all cytological criteria are fulfilled were excluded.

Our results correspond to investigations that studied the correlations between FNAC based predictions of tumor classification and subsequent histologic diagnosis.

Correlations between 62 % and 100 % have been described. The highest levels of agreement were documented for squamous cell carcinoma, SCLC and adenocarcinoma [7, 21, 22].

Secondly, with the advent of selective chemotherapeutics the distinction of adenocarcinomas and squamous cell carcinomas became more and more important. This was accomplished by immunohistochemistry as a powerful tool for differentiation of unclassifiable cases that in the past were often defined as NSCLC-not otherwise specified (NSCLC-NOS) [3, 23].

Another potential contributing factor to our low levels of accuracy with regard to adenocarcinomas is the fact that we did not use immunohistochemistry for further differentiation as for surgical decision making a proof or an exclusion of NSCLC was sufficient.

It also has to be kept in mind that we did not make use of the NSCLC-NOS class but always strived after a definite diagnosis.

Thirdly, according to several studies in cases of adenocarcinomas the heterogeneity of the tumor is responsible for non coinciding diagnoses rather than cytologic misdiagnoses. It must not be neglected that a fine needle aspiration and its cytologic analysis is always a snap-shot of a small part of the tumor. The histologic processing of the sample, however, can identify different differentiation patterns of the tumor and comes to a diagnostic conclusion respective of its predominant differentiation.

Intraoperative sampling error, though rarely observed, cannot be excluded. This leads to the fact that “the false positive rate of intraoperative cytology should approach zero, similar to frozen section analysis; however, the lack of histologic tissue orientation increases the chance for false negative rate” [24].

Our data corroborate that not only the tumor localization in the lung parenchyma and the harvesting technique influence the degree of sampling error but also to a lesser extent the tissue texture. Thus, these are the factors that account for the sensitivity of FNAC.

On the other hand, cytologic analysis is the most relevant factor for specificity. The extent of specificity, however, is essentially influenced by the composition of the included collective. Our collective with its large number of patients and its broad spectrum of diseases represents a realistic image of potential diagnostic difficulties in daily clinical routine. Additionally, this data base makes it possible to draw conclusions concerning diagnostic limitations when using different bioptic techniques such as EBUS- or EUS-FNAC.

Nevertheless it must be acknowledged that in contrast to bronchoscopically harvested specimens, our surgical specimens represent a selection of undistinguishable tumors whose dignity could obviously not be clarified by means of minimally invasive procedures so that a surgical evaluation had to follow. As a matter of fact, SCLC were underrepresented and adenocarcinomas were overrepresented in these collectives.

Our series documented only one chronic inflammatory process that was falsely judged cytologically to be malignant and one case of lipomatous atrophic thymic tissue that was falsely judged cytolgically suspicious of tumor. These findings emphasize cytology’s well-known limitations with respect to mesenchymal neoplasms and highly heterogeneous tumors.

The error of cytologic false-positive diagnoses of cancer is generally based on the overinterpretation of only a few atypical cells.

At the same time it should be emphasized, as shown by Pedio et al. [25], that mesenchymal tumors or lesions of suspicious dignity that are difficult to assess cytologically are also difficult to diagnose histologically.

Sensitivity and specificity are only part of the broader concept of diagnostic accuracy in cytology. This does not only entail the assessment of presence or absence of cancer in a given specimen but also the prediction of cancer differentiation and the identification of benign disease states. The difficult area in which cancer can neither be diagnosed conclusively nor excluded is the area in which frozen section is irreplaceable.

Advanced investigations on the ultrastructure of lung tumors have strengthened the importance of cytology. Bibbo et al. elaborated on this issue stating that in some situations cytologic interpretation is “more accurately reflective of the nature of the lesion than the tissue examined” [26]. This has been supported by several studies identifying a significant cellular heterogeneity present in most of NSCLC and mesenchymal neoplasms. This holds particularly true for large cell carcinoma and poorly differentiated carcinomas regardless if of adenomatous or squamous differentiation [27, 28].

## Conclusions

Intraoperative fine needle aspiration cytology is a valuable, low invasive, time and cost saving diagnostic tool to define the diagnosis of unexplained lung lesions. The degree of differentiation, growth pattern and/or heterogeneity of different lung cancer types determined the reliability of cytological diagnosis. Lung cancer typing according to cytologic criteria is feasible and accurate as well as comparable with results of histologic analysis on small biopsy specimens.

## Abbreviations

EBUS, endobronchial ultrasound; EGFR, epidermal growth factor receptor; EUS, endoscopic ultrasound; FNAC, fine needle aspiration cytology; NSCLC, non small cell lung cancer; NSCLC-NOS, non-small cell lung cancer - not other classified; SCLC, small cell lung cancer; TBNA, transbronchial needle aspiration

## References

[1] Travis WD, Rekhtman N, Riley GJ, Geisinger KR, Asamura H (2010). Brambilla et al. Pathologic diagnosis of advanced lung cancer based on small biopsies and cytology. A paradigm shift. J Thorac Oncol.

[2] Travis WD, Brambilla E, Burke AP, Marx A, Nicholson AG (2015). WHO Classification of Tumours of the Lung, Pleura, Thymus and Heart.

[3] Nicholson AG, Gonzales D, Shah P, Pynegar MJ, Deshmukh M, Rice A (2010). Refining the diagnosis and EGFR status of non-small cell carcinoma in biopsy and cytologic material, using a panel of mucin staining, TTF-1, cytokeratin 5/6 and P63 and EGFR mutation analysis. J Thorac Oncol.

[4] Mok TS, Wu YL, Thongprasert S, Yang CH, Chu DT, Saijo N (2009). Gefitinib or carboplatin-paclitaxel in pulmonary adenocarcinoma. N Engl J Med.

[5] Annema JT, Versteegh MI, Veseliç M, Voigt P, Rabe KF (2005). Endoscopic ultrasound added to mediastinoscopy for preoperative staging of patients with lung cancer. JAMA.

[6] Sharafkhaneh A, Baaklini W, Gorin AB (2003). Yield of transbronchial needle aspiration in diagnosis of mediastinal lesions. Chest.

[7] Welker L, Akkan R, Holz O, Schultz H, Magnussen H (2007). Diagnostic outcome of two different CT-guided fine needle biopsy procedures. Diagn Pathol.

[8] Langer CJ, Besse B, Gualberto A, Brambilla E, Soria JC (2010). The Evolving Role of Histology in the Management of Advanced Non–Small-Cell Lung. J Clin Oncol.

[9] Fischer AH, Cibas ES, Howell LP, Kurian EM, Laucirica R, Moriarty AT (2011). Role of Cytology in the Management of Non–Small-Cell Lung Cancer. J Clin Oncol.

[10] Wallace MB, Pascual JM, Raimondo M, Woodward TA, McComb BL, Crook JE (2008). Minimally invasive endoscopic staging of suspected lung cancer. JAMA.

[11] Annema J, Welker L, Veseliç M and Rabe KF. EUS Imaging and FNA of Primary Lung Tumours. Chapter 12 in Bhutani MS and Deutsch JC. EUS Pathology with Digital Anatomy Correlation. Shelton, Connecticut: People’s Medical Publishing House-USA; 2000. p. 129-135.

[12] Edwards SL, Roberts C, McKean ME, Cockburn JS, Jeffrey RR, Kerr KM (2000). Preoperative histological classification of primary lung cancer: accuracy of diagnosis and use of the non-small cell category. J Clin Pathol.

[13] Schreiber G, McCrory DC (2003). Performance characteristics of different modalities for diagnosis of suspected lung cancer: summary of published evidence. Chest.

[14] Zakowski MF (2003). Pathology of small cell carcinoma of the lung. Semin Oncol.

[15] Wallace WAH, Rassl DM (2011). Accuracy of cell typing in nonsmall cell lung cancer by EBUS/EUS–FNA cytological samples. Eur Respir J.

[16] Xu X, Chung JH, Jheon S, Sung SW, Lee CT, Lee JH (2010). The accuracy of frozen section diagnosis of pulmonary nodules: evaluation of inflation method during intraoperative pathology consultation with cryosection. J Thorac Oncol.

[17] Marchevsky AM, Changsri C, Gupta I, Fuller C, Houck W, McKenna RJ (2004). Frozen section diagnoses of small pulmonary nodules: accuracy and clinical implications. Ann Thorac Surg.

[18] Hwang TS, Ham EK, Kim CW, Chi JG, Park S (1987). An Evaluation of Frozen Section Biopsy in 4434 Cases. J Korean Med Sci.

[19] Orki A, Tezel C, Kosar A, Ersev AA, Dudu C, Arman B (2006). Feasibility of Imprint Cytology for Evaluation of Mediastinal Lymph Nodes in Lung Cancer. Jpn J Clin Oncol.

[20] Welker L, Galle J, Vollmer E (2004). Bronchological bioptic diagnosis of lung cancer -- cytology and/or histology?. Pneumologie.

[21] Bonfiglio T, Johnston WW (1983). Transthoracic thin needle aspiration biopsy. Masson Series in Diagnostic Cytopathology.

[22] Dahlgren SE (1967). Aspiration biopsy of intrathoracic tumours. Acta Pathol Microbiol Scand [B].

[23] Rossi G, Papotti M, Barbareschi M, Graziano P, Pelosi G (2009). Morphology and a limited number of immunohistochemical markers may efficiently subtype non-small-cell lung cancer. J Clin Oncol.

[24] Schwartz AM, Henson DE (2007). Diagnostic surgical pathology in lung cancer. Chest.

[25] Pedio G, Landolt-Weber U. Die zytologische Diagnose von Weichteiltumouren Möglichkeiten und Grenzen der Methodik. GBK Gesellschaft zur Bekämpfung der Krebskrankheiten Nordrhein-Westfalen e.V. Düsseldorf Fortbildung aktuell. 1992;60/61:23–27.

[26] Bibbo M, Wilbur D. Respiratory Tract. In: Comprehensive Cytopathology. 3rd ed. Philadelphia: Saunders, Elsevier Inc; 2008. p. 1136.

[27] Albain KS, True LD, Golomb HM, Hoffman PC, Little AG (1985). Large cell carcinoma of the lung: Ultrastructural differentiation and clinicopathologic correlations. Cancer.

[28] Horie A, Ohta M (1981). Ultrastructural features of large cell carcinoma of lung with reference to the prognosis of patients. Hum Pathol.

